# Supplementary material for: Impact of Perineural Invasion and Preexisting Type 2 Diabetes on Patients with Esophageal Squamous Cell Carcinoma Receiving Neoadjuvant Chemoradiotherapy
Source: Cancers (Basel). 2023 Feb 9;15(4):1122. doi: 10.3390/cancers15041122 (PMC9954405; doi:10.3390/cancers15041122)
Supplement: Supplementary file 1 [file cancers-15-01122-s001.zip › cancers-2192869-supplementary.pdf]

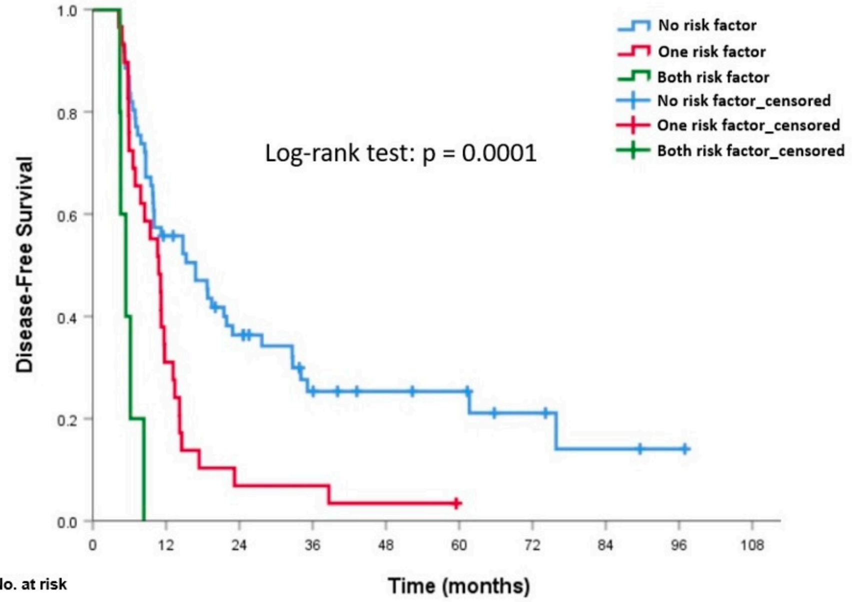

No. at risk									
No risk factor	61	32	20	11	8	7	4	2	1
One risk factor	29	9	2	1	1				
Both risk factor	5	0							

Supplementary Figure S1. Kaplan–Meier curve of disease-free survival for comparison among patients with no risk factors, one risk factor, and both risk factors in the non-pCR patient subgroup. Abbreviation: pCR: pathological complete response.

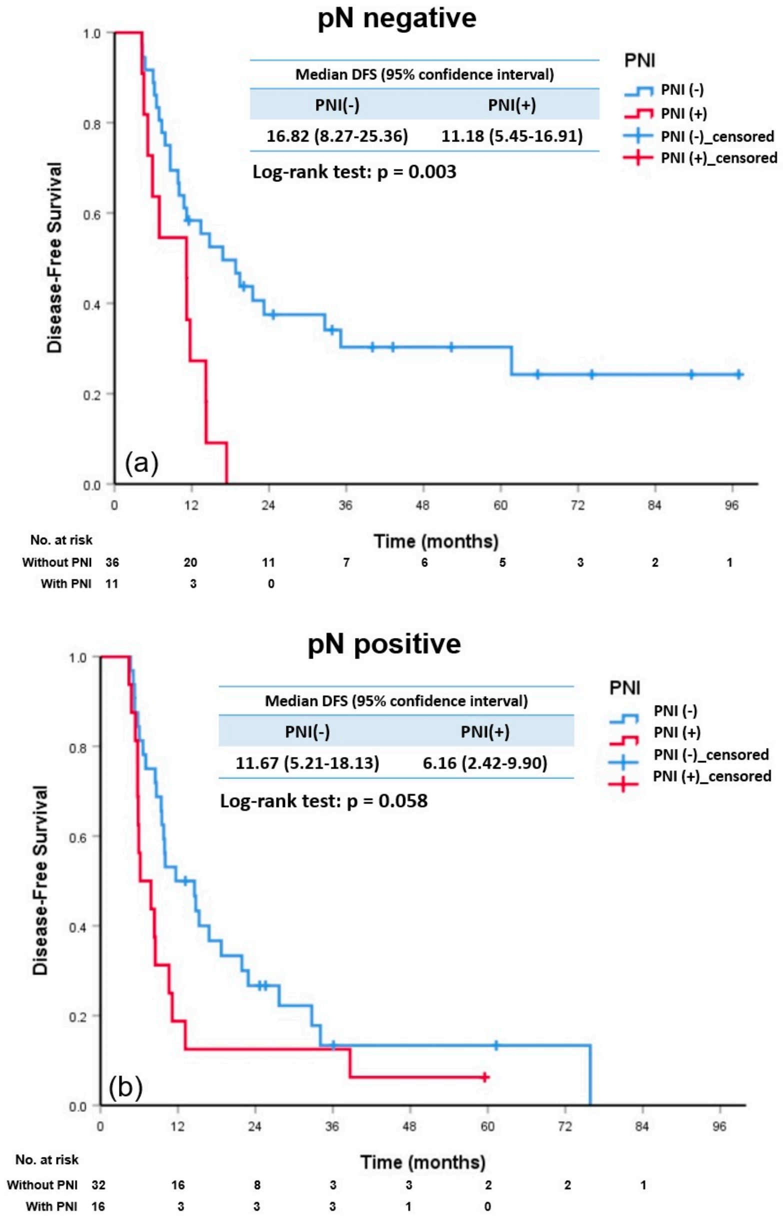

Supplementary Figure S2. Kaplan–Meier curve of DFS for comparison between the presence and absence of PNI in the subgroup of (a) node-negative patients and (b) node-positive patients.

Abbreviations: DFS: disease-free survival; PNI: perineural invasion; pN: pathological lymph node
